# Considerations for supporting meaningful stakeholder engagement in global mental health research

**DOI:** 10.1017/S204579602200035X

**Published:** 2022-07-20

**Authors:** Jill K. Murphy

**Affiliations:** Department of Psychiatry, Mood Disorders Centre, University of British Columbia, Vancouver, BC, Canada

**Keywords:** Evidence-based psychiatry, mental health, multicultural, research design and methods

## Abstract

The need to ensure that research evidence is adopted by health systems and is informed by lived experience expertise has been increasingly recognised in mental health research. In the field of global mental health (GMH), though some progress has been made, the meaningful engagement of key stakeholders in research remains low. This editorial outlines recommendations to support the meaningful engagement of policy makers and people with lived or living experience of mental illness in GMH research. Recommendations include: increasing funding structures that are designed to support meaningful engagement; urging institutions to consider administrative structures that support engagement with lower resourced partners; promoting capacity development opportunities and resources to support researchers to promote meaningful engagement; developing research governance structures that include key stakeholders; and, taking steps to ensure the needs of diverse stakeholders are met through their engagement in research. Examples of good practice from these areas are provided. Though not an exhaustive list of recommendations, this editorial represents a call to the GMH research community to take a deliberate and proactive approach to prioritising meaningful stakeholder engagement in GMH research with the ultimate goal of improving accessible and appropriate mental health care.

## Introduction

A long-recognised challenge in mental health research (as in all fields of health research) is bridging the ‘know-do’ gap, referring to the gap between what we know (research evidence) and what we do (practice and policy). Similarly, the need to ensure that mental health policies and practice are appropriate and acceptable for people with lived or living experience (PWLE) of mental illness has been increasingly acknowledged (Ennis and Wykes, 2013). These priorities reflect the recognition that efforts to ensure that evidence-based policies and practice (EBP) reach those in need should be integral to the research endeavour. In mental health in particular, availability and accessibility of care is often limited and approaches to mental health care may not be appropriate or patient centred.

In recent years, there has been an emergence of approaches to promote the uptake of research findings by health systems. Implementation science is identified as essential to understanding the mechanisms and processes that promote the real world uptake, implementation and scale-up of EBP (Betancourt and Chambers, 2016; Murphy *et al*., 2022). Approaches that promote the engagement and collaboration of key stakeholders in research are also increasingly prioritised. Integrated Knowledge Translation (Kothari *et al*., 2017) (iKT) is an emerging field whereby ‘knowledge users’ (e.g. policy makers, clinicians) are engaged throughout the research process with the goal of promoting evidence-based policy and practice (Boland *et al*., 2020). Community-based Participatory Research (CBPR) (Smikowski *et al*., 2009) and Patient-Oriented Research (POR) (Johnston *et al*., 2021) involve the active participation of PWLE and their communities at all stages of research to ensure that research addresses the priorities of PWLE and ultimately leads to improved experiences of health care.

There is a clear call to action to better integrate policy and practice with evidence and lived experience expertise. Yet, several gaps in the implementation of these approaches exist. Global mental health (GMH) is an interdisciplinary field aiming to promote mental health equity via the prevention, care and treatment of mental illness and the promotion of mental well-being worldwide (Collins, 2020). GMH focuses on addressing mental health equity challenges, which are often particularly evident in low- and middle-income countries (LMICs) and/or among historically marginalised populations. Given the emphasis on equity, increased and enhanced engagement with key stakeholders is necessary in GMH to promote scale-up of EBP and to ensure policy and practice is responsive to the needs of PWLE in underserved contexts. Despite claims that a shift towards the inclusion of PWLE in GMH is underway (Patel *et al*., 2018), in a 2019 review (Ryan *et al*., 2019) of service user involvement in GMH, authors found that of the ten papers identified that described service user involvement in mental health systems strengthening, only one engaged PWLE in the research process. A previous systematic review found no examples of service user engagement in GMH research (Semrau *et al*., 2016). Engagement with policy makers is also a challenge in GMH. In a qualitative study of barriers and drivers to stakeholder engagement in GMH (Murphy *et al*., 2021) studies, though engagement with policy makers was recognised as a key component of GMH implementation research (and indeed is often required by funding agencies), researchers reported many challenges related to engaging policy makers in a meaningful way.

This editorial introduces considerations for the engagement of policy makers and PWLE in GMH research, outlining recommendations that may support meaningful engagement. Stakeholder engagement in research is of course not limited to these two groups and may involve clinicians, community and faith-based organisations, the media, representatives of other sectors including education and social services and many more (Murphy *et al*., 2021). A discussion of engagement with all potential stakeholder groups is beyond the scope of this paper, though some themes discussed herein will be applicable across stakeholder groups. In this paper, I use the term PWLE to refer to stakeholders who have lived or living experience of mental illness. PWLE may also be referred to as ‘service users’, ‘end users’, ‘patients’ and ‘consumers’ among other terms. Definitions of ‘meaningful engagement’ in the literature are varied (Black *et al*., 2018; Tricco *et al*., 2018; Goldstein *et al*., 2021). For the purpose of this paper ‘meaningful engagement’ refers broadly to the engagement of stakeholders at all stages of the research process, where engagement is not tokenistic but instead consists of partners playing a role where they are actively engaged (e.g. via joint priority setting, co-design, collaboration in knowledge exchange activities, etc.), and are appropriately acknowledged and compensated for their participation.

## Background

Stakeholder engagement is recognised as crucial by GMH researchers, but challenges and barriers are persistent. In a qualitative study examining barriers and drivers of several components of GMH implementation among a portfolio of researchers funded by Grand Challenges Canada (GCC) (Endale *et al*., 2020; Esponda *et al*., 2021; Murphy *et al*., 2021; Qureshi *et al*., 2021), we asked study participants to self-select which topics they preferred to focus on in interviews. Though we did not quantify types of responses, a majority of study participants chose to speak about stakeholder engagement in their interviews, describing the process as both essential and oftentimes difficult. Several challenges were described, the majority of which highlighted the barriers to achieving active or meaningful collaboration with study stakeholders. Notably, the nature of engagement of PLWE described by study participants generally related to service uptake within the context of research studies, with little discussion of engaging PWLE in the research process itself. Challenges related to engaging with policy makers also included achieving meaningful engagement, with the gap between ‘on paper’ collaboration and active participation by policy makers highlighted by several participants. Communication and dissemination of results in a way that is appropriate for the policy context was also identified as a barrier among researchers. Overall, the results of the study suggest that meaningful engagement of PWLE and policy stakeholders in GMH remains elusive and difficult. This is consistent with previous research about the involvement of PWLE in GMH research (Semrau *et al*., 2016, Ryan *et al*., 2019) as described above. Though engagement of PWLE in GMH research may have increased somewhat since the publication of these studies, the gap in this area remains evident. Though policy considerations for GMH are frequently outlined in the literature, there is a dearth of literature detailing approaches to policy maker engagement in GMH research.

## Recommendations to support meaningful stakeholder engagement

This gap in meaningful engagement of policy makers and PWLE stakeholders in GMH research suggests that concrete steps are necessary to support and promote these processes. Research funding agencies, universities, research networks and other bodies can provide support in the form of tailored funding, structural support, capacity development, governance structures and ensuring stakeholder needs are met to facilitate enhanced and meaningful engagement in GMH research. Below, I offer some recommendations and examples of good practice to encourage increased support for meaningful stakeholder engagement.

1. Funding Opportunities Designed to Support Meaningful Engagement

Funding agencies play a key role in setting the agenda for GMH research, including the processes undertaken by funded researchers. Increasingly, stakeholder engagement is central to funding calls in GMH, with requirements for evidence of partnerships with policy makers, community partners and other stakeholders embedded in calls for proposals. GMH implementation science funders, including GCC and the Global Alliance for Chronic Diseases (GACD), recognise that engagement with key stakeholders is fundamental to the success of implementation science research; this represents a positive step in promoting stakeholder engagement in GMH research. There are several additional actions that funding agencies could take to promote and support meaningful and long-term engagement in research. In our qualitative study about stakeholder engagement in GMH implementation, time, trust and deep contextual understanding were identified as core facilitators of engagement (Murphy *et al*., 2021). Several participants expressed frustration with trying to establish and maintain collaborative relationships during two or three-year funding cycles. These short funding cycles create a barrier to building trust and good faith with stakeholders, particularly with groups who are historically marginalised such as Indigenous communities. Funding agencies could therefore support meaningful engagement by creating funding mechanisms that are conducive to the time and trust-building needed to foster meaningful and long-term engagement. This could consist of longer funding cycles (the GACD and its partner agencies already offer five-year implementation science grants), or of dedicated funding pools to support relationship-building and priority-setting activities that convene stakeholders prior to the research funding proposal submission. Support for high-quality formative research (e.g. situational analysis (Murphy *et al*., 2019*a*), stakeholder analysis (Makan *et al*., 2015)) to promote comprehensive contextual understanding is also essential to promote robust implementation science and meaningful engagement.

A collaboration between researchers in Canada, Vietnam and Australia to support enhanced community-based depression care and mental health policy development in Vietnam serves as an example of meaningful policy engagement that has been facilitated by a continuum of support from a funding agency. In 2014 our team received seed funding from GCC to conduct a feasibility study to explore conducting a randomised controlled trial (RCT) of a supported self-management (SSM) intervention for depression in Vietnam (Murphy *et al*., 2018). This work built on a history of mental health policy engagement conducted by colleagues at the University of Melbourne and an established relationship between Simon Fraser University in Canada with the Institute of Population, Health and Development, the key research partner organisation in Vietnam. It also leveraged a policy commitment by the Government of Vietnam (GoV) to enhance community-based depression care in the country. Following the successful pilot study we were funded by GCC to conduct a full-scale RCT in collaboration with the GoV via the Ministry of Invalids, Labour and Social Affairs (MOLISA), who provided matched funding for this study (Murphy *et al*., 2020). This successful RCT and collaboration with MOLISA laid the ground work for additional ongoing studies, funded by GCC and the Canadian Institutes of Health Research (CIHR), to support the adaptation of the intervention for app-based delivery and to support several aspects of mental health policy and system strengthening. The process and history of policy engagement is described in depth elsewhere (Murphy *et al*., 2019*b*). In brief, the continued funding for this study, from pilot to RCT to adaptation and scale-up, in addition to the alignment with GoV priorities and the trust built over time between study team members and policy makers, has led to a collaboration that has been active for almost a decade.

1. Structural Support for Meaningful Engagement

Research structures are also important to support meaningful engagement with stakeholders. GMH engagement often includes partners from low-resource settings and/or representing underserved communities. Research structures such as payment processes and administrative requirements by funding agencies and universities may be prohibitive to partners from organisations or communities with little or no access to core funding or infrastructure support. Policies requiring reimbursement, often with a long delay, of community research partners or organisations, including government agencies, based in LMICs for activities such as travel and honoraria can create a substantial burden for partners. Overly burdensome reporting and administrative requirements can also be prohibitive for smaller or lower-resourced organisations. Though monitoring and accountability by funding agencies and universities is certainly essential, considerations for collaboration with partners from LMICs or lower-resourced communities in high-income settings could help facilitate meaningful and rewarding engagement with both PWLE and policy makers, making engagement in research more appealing.

1. Capacity Development to Support Meaningful Engagement

There is also an evident need for capacity development for researchers to support meaningful stakeholder engagement. Funding agencies and research networks can support these efforts in several ways. Direct capacity-building opportunities through workshops, conferences, short courses and other models can provide excellent support to researchers. For example, the GACD holds an annual Implementation Science School for trainees and early career researchers, and holds a condensed Implementation Science Workshop for all members of its research network as part of its annual scientific meeting. Similar approaches could support capacity building for specific research approaches and methodologies such as iKT, CBPR, POR and communication and dissemination strategies (e.g. policy briefs, plain language summaries) to reach different stakeholder groups. Funding agencies may also develop resources and training to support stakeholder engagement. For example, the CIHR has developed a ‘Guide to Knowledge Translation Planning at CIHR: Integrated and End-of-Grant Approaches’ (https://cihr-irsc.gc.ca/e/documents/kt_lm_ktplan-en.pdf) to support researchers to plan for and implement all aspects of knowledge translation including iKT. Funding agencies may also support networks focused on aspects of stakeholder engagement that can provide capacity-building opportunities and guidance for researchers. For example, as part of its Strategy for Patient-Oriented Research (SPOR), the CIHR has collaborated with provincial and territorial research funding bodies to fund nine SPOR networks across Canada. These networks provide specialised services to researchers, clinicians, PWLE, policy makers and others to support patient-oriented health research.

Specific to engagement with PWLE, approaches for partnering appropriately with diverse and potentially historically marginalised groups must be considered. Many communities live with the legacies of colonialism, experience ongoing racism, discrimination and marginalisation. The British Columbia (BC) SPOR network (‘the BC Support Unit’, https://www.bcahsn.ca/our-units/bc-support-unit) is providing leadership in this area. The BC SUPPORT Unit's Patient Engagement Methods Cluster has created a suite of educational modules designed to support the inclusion of diverse patients in health research. The modules were created in a collaboration between patient partners and academic researchers, with the aim of providing relevant context and practical tools for engaging in research with more diverse PWLE. The first module is a primer to diversity in patient engagement, and the remainder focus on a series of historically marginalised communities: LGBTQ2S+, rural and remote, disabled, d/Deaf and immigrant, refugee, racialised and ethnocultural. The modules are presented using an interactive educational platform called the Tapestry tool and are available at: https://sites.tapestry-tool.com/bcsupport/tapestry/bc-support-unit-tapestry-project/#/nodes/1615. These tools can help to inform diverse partnerships in GMH research, and similar capacity-building resources could be created by relevant GMH networks to provide guidance for meaningful engagement with PWLE in diverse settings, including LMICs. The Research on Equity in Mental Health in the Asia Pacific-Digital (REMAP-D) international research excellence cluster, hosted at the University of British Columbia (UBC), is currently creating similar modules, one of which will explore considerations for equitable engagement with PWLE in GMH.

1. Including Stakeholders in Research Governance

Engagement of policy makers and PWLE in all aspects of the research process is fundamental to fostering meaningful engagement in research. The inclusion of stakeholders in the governance structures of research networks or teams is also an approach that promotes meaningful engagement. As part of the previously-described programme of research in Vietnam, we have a Project Advisory Committee that includes policy maker partners. The REMAP-D research cluster includes an International Lived Experience Advisory Council (I-LEAP) as part of its core governance structure. The mandate of the I-LEAP is to consult on all of REMAP-D's activities, providing lived experience expertise to inform priority setting for research and knowledge exchange activities and ensuring that the purview of the cluster aligns with priorities of PWLE from across the Asia Pacific region. The I-LEAP governance structure builds on established leadership in the field of patient-engaged research by the The Collaborative RESearch Team to study psychosocial issues in Bipolar Disorder (CREST.BD, www.crestbd.ca/), an international research network based at UBC that includes people living with bipolar disorder in all aspects of their research and governance. Existing networks led by PWLE, such as the Global Mental Health Peer Network, provide leadership for mental health priority setting and advocacy worldwide. These networks also participate in research and represent important connections between research and mental health advocacy.

1. Considering Context and Stakeholder Needs

Finally, it is important to acknowledge that GMH research often takes place in challenging contexts which may be contending with the effects of conflict or natural disasters, minimal financing for mental health in the health sector budget, low prioritisation of mental health by international funding agencies, high levels of mental illness stigma and discrimination and numerous competing priorities. The coronavirus disease 2019 (COVID-19) pandemic has likely compounded these challenges, though the need for enhanced mental health care has also been highlighted by the pandemic (Maulik *et al*., 2020; Kola *et al*., 2021). Engaging with PWLE who may be experiencing economic hardship means that engagement may create a burden for partners if they are not adequately compensated. PWLE also have busy lives and obligations, meaning that they must be supported to participate in a way that is appropriate for their lives (e.g. work hours, child care responsibilities, etc.) These factors can create further challenges and must of course be considered when planning for stakeholder engagement. Formative research and consultation processes that prioritise listening and trust building along with flexibility and humility by researchers are therefore essential to supporting engagement.

## Conclusion

The benefits of engaging key stakeholders, including policy makers and PWLE, in GMH research are evident. Meaningful stakeholder engagement increases the likelihood that research will lead to real impact for health systems, communities and individuals (Ennis and Wykes, 2013). There is also an ethical imperative to meaningfully engage appropriate stakeholders in research that will directly impact them. The *Lancet Commission on global mental health and sustainable development* (Patel et al., 2018) uses the term, originating in the disability rights movement, ‘nothing about us without us’ to describe a shift in GMH toward the engagement and empowerment of PWLE in all aspects of mental health policy and care and in their own recovery. This shift must also take place in the research community, with a commitment by funding agencies, research institutions, networks and individual researchers to promote meaningful and active engagement by key stakeholders. This commitment requires concrete steps that may be facilitated by those driving the research agenda and adopted by researchers themselves. It also requires a commitment to ensuring that engagement in research is beneficial and positive for key stakeholders, which may require a shift in the status quo of how research is conducted, necessitating humility, openness and creativity on the part of researchers.

The recommendations and examples provided in this paper are by no means exhaustive, but provide an overview of actions that can be taken by the GMH research community to support meaningful engagement by policy makers, PWLE and other stakeholders in research. More research regarding approaches and methodologies that engage stakeholders in research are also needed, and will provide a much needed contribution to field of GMH.
